# Novel nomograms for predicting the risk of low distal bone strength: development and validation in a Chinese population-based observational study

**DOI:** 10.1186/s13018-023-03546-6

**Published:** 2023-01-30

**Authors:** Congzi Wu, Ting Liu, Zhenyu Shi, Liang Fang, Hongting Jin, Peijian Tong

**Affiliations:** 1 Institute of Orthopaedics and Traumatology, The First Affiliated Hospital of Zhejiang Chinese Medical University, Hangzhou, 310053 Zhejiang Province People’s Republic of China; 2 The First College of Clinical Medicine, Zhejiang Chinese Medical University, Hangzhou, 310053 Zhejiang Province People’s Republic of China; 3 School of Nursing, Zhejiang Chinese Medical University, Hangzhou, 310053 China; 4 Department of Orthopaedic Surgery, The First Affiliated Hospital of Zhejiang, Chinese Medical University, Hangzhou, 310003 Zhejiang Province People’s Republic of China

**Keywords:** Bone strength, Nomogram, Prognostic model, Quantitative ultrasound measurement (QUS)

## Abstract

**Background:**

This study aims to develop nomogram models based on the speed of sound (SOS) measurements results along with demographic information to predict the risk of low bone strength (LBS) of radius appropriate to the Chinese population of a broad age spectrum.

**Methods:**

A population-based cross-sectional study was conducted in 5 outpatient clinics located in Zhejiang, the southern part of China. A total of 38,699 participants from 2013 to 2017 were included. Baseline measurements included SOS of the distal radius and clinical risk factor evaluation. Logistic regression models were used to evaluate prognosis and identify independent predictive factors, which were then utilized to establish nomograms for predicting the low bone strength of radius. The discrimination and calibration of nomograms were validated using the calibration plots, the decision curve analysis (DCA), and the receiver operating characteristics curve (ROC).

**Results:**

A total of 19,845 of the 38,904 participants ranged in age from 10 to 88 years were selected in this process. LBP nomogram model 1 was constructed based on age, weight, height, BMI, and gender. LBP nomogram model 2 was constructed based on age, height, BMI, and gender. The AUCs for model 1 and model 2 were 0.838 (95% CI: 0.832–0.844) and 0.837 (95% CI: 0.831–0.843), respectively. High-quality calibration plots and DCA in nomogram models were noticed, indicated that the constructed nomogram models were clinically useful.

**Conclusions:**

Our study demonstrates that the nomograms established in this study could effectively evaluate the high-risk population groups of distal radius fracture in China.

## Introduction

Bone strength is determined by its material and structural properties. Bone mineral density (BMD) is a useful tool for diagnosis. However, this parameter provides information regarding only the quantity of mineral in bone, which is only one component of bone strength [1]. Since bone density does not fully explain the variance in bone strength, it has been suggested that the ability to provide structural information may improve the estimation of bone strength and fracture risk [2, 3].

Quantitative ultrasound (QUS) is a mechanical wave that can be influenced by BMD in addition to bone structure, which are equally important in determination of bone strength [3, 4]. QUS has the advantages of being free of radiation, easy to use, portable, and lower in cost than dual-energy X-ray absorptiometry (DXA) [5] that may aid the identification of individuals at risk for fracture in a primary or secondary care setting, and is also used for long-term monitoring of bone health or integrity [5]. Speed of sound (SOS) is one parameter of skeletal status provided by QUS assessment, and the significance of the low SOS parameters in the fact that it is a signal being predictive of deficient bone strength and future fracture risk [6]. There is ample evidence documenting the ability of SOS which provides information on bone microarchitecture and material properties to assess bone strength, and it is partly independent of BMD and clinical risk factors in predicting fractures [7–9].

Bone strength of radius has important clinical significance for assessing local bone mass and screening high-risk patients with distal radius fractures [10]. There have been many studies focused on comparing the predictive power of SOS for fractures [5, 11, 12]. Several researches have shown that radius SOS data not only has a significant correlation with the risk of distal radius fractures [6], but also has a potential correlation with age, gender, and body mass index (BMI) [8, 13]. However, the sample size of these studies is relatively small, which is weak evidence for revealing the clinical value of SOS. Therefore, this study collected radial SOS data and demographic information (including height, weight, body mass index, etc.) of community residents from multiple areas in Zhejiang Province. We performed regression analysis on an all-age cohort (10–88 years old) including 38,904 participants and established a validated nomogram prediction model, aiming to explore the influencing factors of bone strength of radius and establish a low bone strength (LBS) of radius prediction model, and expected to provide a new auxiliary method for clinical assessment of the risk of distal radius fractures.

## Materials and methods

### Study design and participants

This cross-sectional study is a sub-cohort analysis of a study on SOS characteristics at the outpatient clinics of public health organizations in Zhejiang Province of China, including Hangzhou, Wenzhou, Ningbo, Jiaxing, Huzhou, between June 2013 and November 2017, using a total of 38,904 participants aged 10–88 [13]. All participants completed a standardized questionnaire before their scan (including age, medical history, etc.). Height, weight, and body mass index (BMI, kg/m^2^) were measured. Study exclusion criteria included metabolic and endocrine diseases; bone tumors; renal insufficiency; secondary causes of osteoporosis, such as Cushing’s disease, hyperthyroidism, Crohn’s disease, or rheumatoid arthritis; and use of a bone active agent or hormone therapy within half a year. All participants offered informed consent, and this study was approved by the Institutional Review Board of The First Affiliated Hospital of Zhejiang Chinese Medical University (IRB No. 2018-ZX-02601).

### Ultrasound measurement

The study employed the Sunlight Mini-Omni Ultrasound Bone Sonometer (Sunlight; BeamMed Ltd., Israel) with 3 different probes allowing for SOS measurements to be recorded at the radius. First, ultrasound gel was applied to the skin surface at the measurement site to facilitate acoustic coupling. And then the handheld probe was placed at the distal radius. The operator rotated the transducers within the probe around the radius slowly without lifting the probe from the skin surface. The measurement procedure was repeated at least 3 times. After the signal is digitized and stored, the data are sent to a computer for automated analysis. All measurements were handled by a single trained operator throughout the study. The QUS measurement was expressed in SOS, which was provided by the instrument. LBS was defined if SOS < 4100 m/s.

### Statistical analysis

IBM SPSS Statistics 25.0 and R 4.0.3 were used for statistical analyses. The chi-square test or Fisher’s exact probability test was used to compare count data. The normal distribution of the data was evaluated by QQ-plot and histogram. Different variables were described by the mean ± SD. When comparing the means between groups, the Student’s t test or one-way ANOVA was used. A *p* value < 0.05 was taken to indicate statistical significance. Logistics regression analysis was used to consider whether the low bone strength of radius was related to age, weight, height, BMI, and gender. Two models were constructed by backward stepwise regression analysis and L1-penalized least absolute shrinkage and selection operator (LASSO) method, respectively. Risk factors were identified using stepwise logistic regression analysis, and the backward stepwise method was used to select the variables that were eventually included in model 1. Besides, the “glmnet” package was used to perform the LASSO regression for variables selection and multivariable analysis, augmented with tenfold cross-validation for internal validation (model 2). Then, nomogram plots were drawn based on regression analysis. To internally validate, the bootstrapping validation (200 bootstrap resamples) was undertaken for both models to calculate a relatively corrected C-index. Calibration plots were used for the comparison between nomogram-predicted and actual outcomes using a 45-degree line as an optimal model. Additionally, decision curve analysis (DCA) was plotted for the threshold probabilities range of nomograms in association with model 1 and model 2. Finally, time-dependent receiver operating characteristic (ROC) curves, including the area under the curve (AUC) and its 95% confidence interval (95% CI), were analyzed by the pROC package to evaluate the performance of prognostic prediction.

## Results

### Demographics of participants

**Fig. 1:**
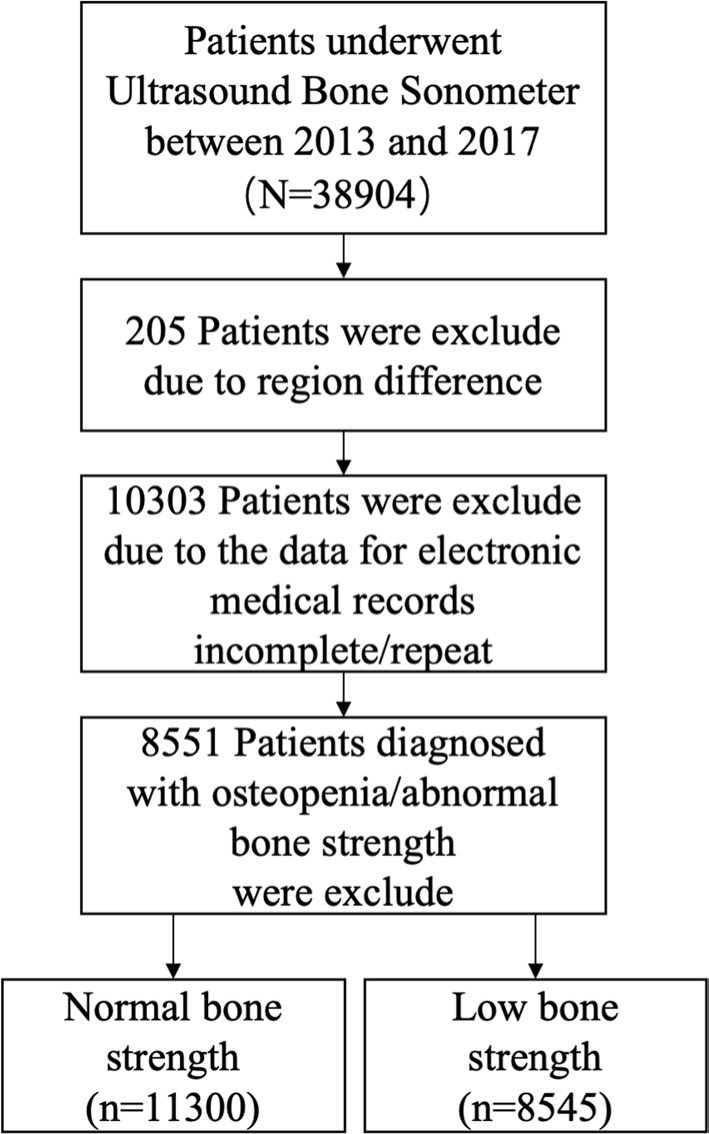
Flow diagram of patient selection and inclusion

A total of 19,845 of the 38,904 participants ranged in age from 10 to 99 years were included in this study, and 19,059 subjects were excluded for reasons. A flow diagram of the inclusion process is demonstrated in Fig. 1.

Baseline characteristics are shown in Table 1. A total of 8545 participants were diagnosed with low bone strength of radius at baseline comprised of 85.7% females with an overall age of 63.71 ± 11.23 years and BMI of 23.31 ± 3.31 kg/m^2^. As expected, patients with LBS were, on average, older and had shorter height, lower body weight, and higher BMI than those with normal bone strength (NBS). Furthermore, all QUS measurement was significantly lower in the LBS group than in the NBS group. In addition, patients with LBS had a higher proportion of women (7324 (85.7%) versus 6687 (59.2%), *p* < 0.001) than controls in gender distribution.

**Table 1:** Characteristics of NBS and LBS individuals

Variables	Total	NBS	LBS	Test statistic	*p* value
No.	19,845	11,300 (56.94%)	8545 (43.06%)		
Region					
Hangzhou	17,279	9797	7482	321.24	< 0.01
Huzhou	26	8	18		
Jiaxing	1853	1275	578		
Ningbo	32	26	6		
Wenzhou	655	194	461		
Sex					
Male	5834	4613	1221	1650.48	< 0.05
Female	14,011	6687	7324		
Age, years	57.96 ± 14.12	52.86 ± 13.94	63.71 ± 11.23	−64.34	< 0.01
< 24	247	182	65	3080.76	< 0.05
25–45	3471	3151	320		
46–64	9381	5623	3758		
65–80	6040	2179	3861		
> 80	706	165	541		
Weight, kg	59.63 ± 9.81	60.83 ± 9.58	58.05 ± 9.87	19.95	< 0.01
Height, cm	160.11 ± 7.26	162.02 ± 6.94	157.58 ± 6.90	44.71	< 0.01
BMI, kg/m^2^	23.20 ± 3.14	23.12 ± 2.99	23.31 ± 3.31	−4.39	< 0.01
< 18.5	1026	510	516	92.03	< 0.01
18.5– 24.9	13,367	7879	5488		
25.0–27.9	3993	2202	1791		
28.0–31.9	1304	641	663		
> 32.0	155	68	87		
SOS, m/s	4011.11 ± 258.76	4207.27 ± 127.54	3751.70 ± 125.74	250.67	< 0.05
T scores	−1.42 ± 2.32	0.35 ± 1.10	−3.76 ± 1.11	258.69	< 0.01
Z scores	−0.165 ± 1.91	1.10 ± 1.26	−1.84 ± 1.23	165.00	< 0.01

*NBS* normal bone strength, *LBS* low bone strength, *BMI* body mass index, *SOS* speed of sound

### Selection of predictive indicators for low bone strength of radius

The multiple stepwise regression analysis showed that 5 variables, including age, weight, height, BMI, and gender, were statistically significant risk factors. So all of these variables were imported to the binary multivariate logistic regression as model 1. The multivariate logistic regression analysis demonstrated that gender (odds ratio (OR) = 1.125, 95% confidence interval (CI) = 1.008–1.254), age (OR = 1.103, 95% CI = 1.099– 1.107), weight (OR = 1.247, 95% CI = 1.189–1.308), height (OR = 8.435, 95% CI = 8.136–8.745), and BMI (OR = 5.939, 95% CI = 5.258–6.706) were independent risk factors for LBS (Table 2).

**Table 2:** Models for predicting low bone strength

Intercept and Variable	Model 1		Model 2	
	Coefficient	Odds Ratio (95% CI)	Coefficient	Odds Ratio (95% CI)
Intercept	20.677		−5.286	
Gender	−2.185	1.125 (1.008 to 1.254)	−2.132	0.118 (0.106 to 0.132)
Age	0.098	1.103 (1.099 to 1.107)	0.097	1.102 (1.098 to 1.106)
Weight	0.221	1.247 (1.189 to 1.308)	NA	NA
Height	−0.170	8.435 (8.136 to 8.745)	−0.006	0.993 (0.987 to 1.000)
BMI	−0.521	5.939 (5.258 to 6.706)	0.038	1.039 (1.028 to 1.050)

*BMI* body mass index

**Fig. 2:**
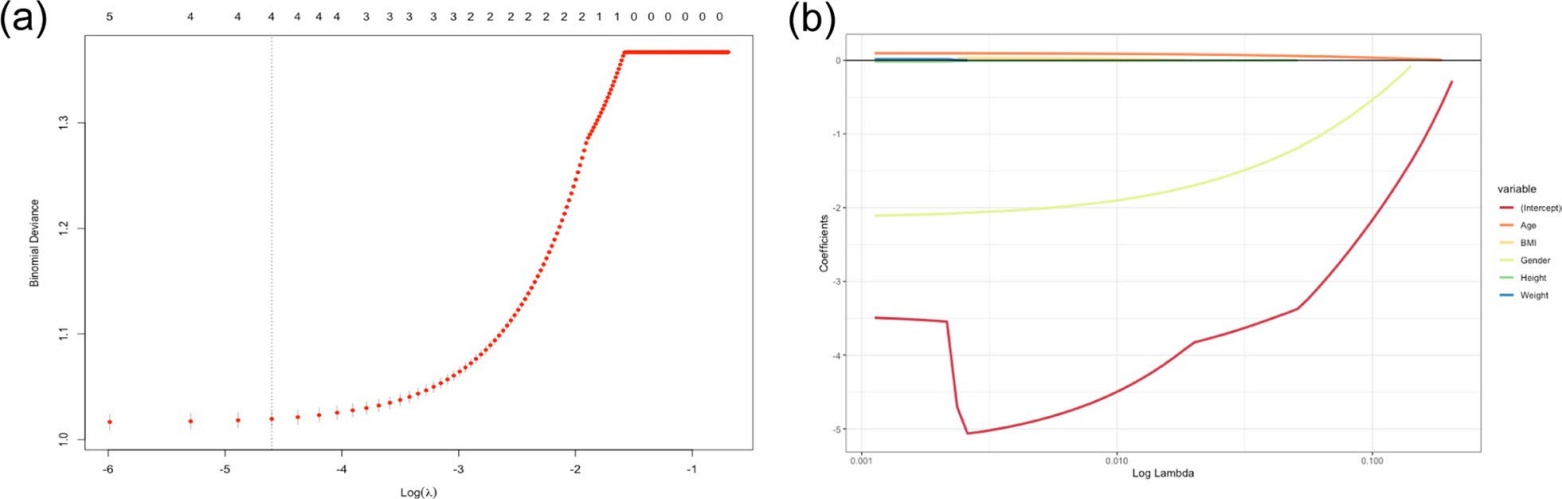
Selection of prognostic factors using the LASSO regression model. **a** LASSO regression used tenfold cross-validation to determine the optimal tuning parameter (*λ*). The partial likelihood deviance was plotted versus log(*λ*). **b** LASSO coefficient profiles of all variables of LBS. Coefficient profiles were observed against the log lambda sequence

However, five variables were reduced to 4 potential predictors with nonzero coefficients after LASSO regression selection based on the analysis of tenfold cross-validation. These predictors included age, height, BMI, and gender (Fig. 2). The above-mentioned indicators were included in the multivariate logistic regression as model 2. The multivariate logistic regression analysis revealed that gender (odds ratio (OR) = 0.118, 95% confidence interval (CI) = 0.106–0.132), age (OR = 1.102, 95% CI = 1.098– 1.106), height (OR = 0.993, 95% CI = 0.987–1.000), and BMI (OR = 1.039, 95% CI = 1.028–1.050) were independent risk factors for LBS.

### Construction and validation of Nomogram

**Fig. 3:**
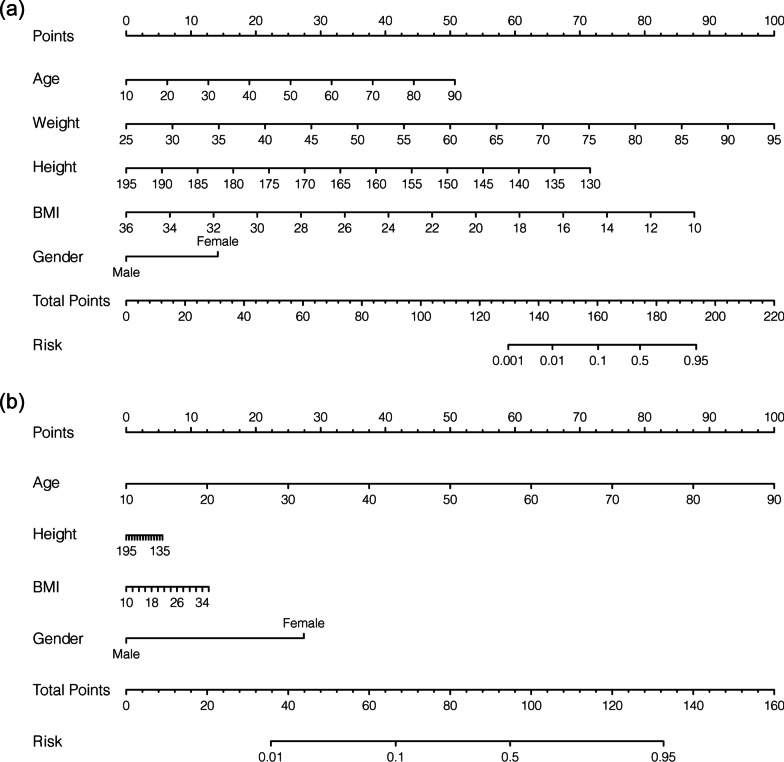
Nomograms for LBS based on model 1 and model 2. **a** Nomograms for LBS in model 1; **b** nomograms for LBS in model 2. LBS, low bone strength

**Fig. 4:**
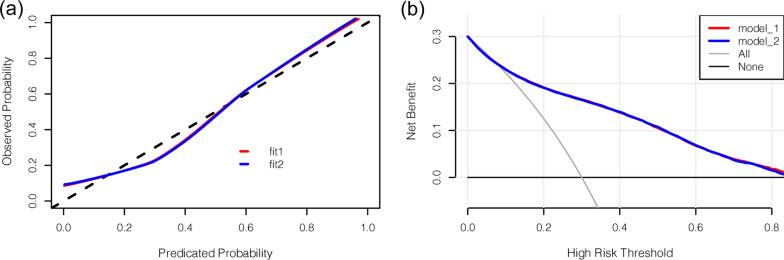
Calibration plots and decision curve analysis (DCA) of nomograms for osteoporosis in both model 1 and model 2. **a** Calibration curves of nomograms in terms of agreement between the predicted risk and actual observed outcomes; **b** decision curve analysis of the nomogram for LBS. LBS, low bone strength

**Fig. 5:**
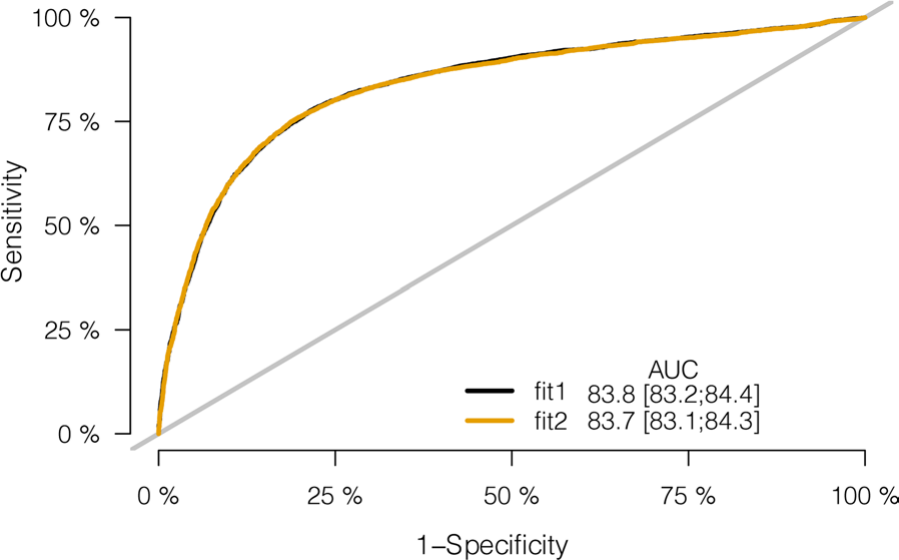
ROCs curve for the nomograms ROC, receiver operating characteristic

Afterward, the nomogram was developed based on model 1 (with age, weight, height, BMI, and gender) or model 2 (with age, height, BMI, and gender) and was used to quantitatively predict the LBS risk (Fig. 3). The C-index of the nomogram was 0.838 (95% CI: 0.832–0.844) for model 1, and 0.837 (95% CI: 0.831–0.843) for model 2, which was identified to be 0.838 via bootstrapping validation in both models (Bootstrap = 200). For orthopedic doctors, the plot was available to locate a patient’s levels of age, weight, height, BMI, and gender in each axis; to draw a line straight upward to the point axis and sum up the total points; and then to draw a line straight down to determine their risk of LBS. The calibration curve illustrated a fair agreement between the predicted probabilities and the observed proportions in both nomograms for model 1 or model 2 (Fig. 4a). On decision curve analysis, the results indicated that using the developed nomograms to predict the LBS probability displayed the better net benefit than either the treat-all or treat-none scheme, indicating that the constructed nomogram models were clinically useful, while nomogram for model 1 and nomogram for model 2 were very similar when the threshold probability is greater than 32% (Fig. 4b). Furthermore, the acceptable AUC values for the ROC curves were also noticed for prediction performance evaluation in model 1 and model 2, respectively (Fig. 5). The AUC for model 1 was virtually identical to that for model 2. However, model 1 with all five factors had a slightly higher AUC value (83.8% ± 0.6%) than model 2 (83.7% ± 0.6%).

## Discussion

As far as radius is concerned, the distal radius fractures (DRFs) are the most common hazard. Epidemiological surveys indicate that DRFs are the most common upper limb fractures in patients over 65 years of age [14], and accounting for 26–46% of all skeletal fractures observed in primary care [15, 16]. Younger patients receive a DRF after an adequate trauma, and elderly patients suffer fractures through low-energy mechanisms. DRFs cause a decline in clinically important functions, which is an important reason for mortality or loss of independence in the elderly population [17, 18]. Low-energy fractures are hallmarks of low bone strength, and DRF patients had a 1.51-fold and 1.40-fold higher incidence of hip fracture and spinal fracture, respectively [10, 19]. Although a fall from a standing height is the most common cause of DRFs, sufficient bone strength can withstand this impact more and reduce the risk of DRFs [20]. Bone strength and quality is determined by bone architecture (including geometry and microarchitecture) and material properties (including mineralization and collagen cross-links) [2, 12]. BMD can provide insights regarding material properties and is a significant predictor of bone strength [1]. Nevertheless, the added value of bone architecture in estimating bone strength should not be ignored [3]. SOS may identify aspects of bone quality not completely captured by BMD, such as microarchitecture or material properties, and can be used for bone strength or integrity assessment [3, 8]. Therefore, identifying the low bone strength of radius through SOS can be used to screen high-risk groups of fractures, especially high-risk groups of DRFs, which can help us to intervene in advance and possibly reduce the incidence, associated morbidity, and health care costs of these injuries.

In this study, we constructed prognostic nomogram models based on age, gender, weight, height, and BMI. All these detected factors are closely related to low bone strength. We found patients with lower bone strength of radius were, on average, older, and had shorter height, lower body weight, and higher BMI than those with normal bone strength, and all SOS measurement was significantly lower in the low bone strength group than in the normal bone strength group. Furthermore, patients with low bone strength of radius had a higher proportion of women (7318 (85.7%) versus 6693 (59.2%), *p* < 0.001) than controls in gender distribution. The two prognostic models were created from the same data source using different statistical methods, and risk factors teased out from one method are not necessarily the same as in another. However, the difference in risk estimates from different models seemed to be minor of clinical concern, since both calibration plots and DCA of the two models aligned almost perfectly. Of note, a high area under the ROC curve (AUC) was noticed for both model 1 and model 2, respectively. The LASSO prognostic model 2 excluded weight and did not perform any better on predicting bone strength of radius. A possible reason for this is that weight was underestimated in the population studies used to develop the models. Additionally, weight might serve as a predictor operating along with other relevant risk factors such as BMI independently, producing a compounding effect on increasing the accuracy of prediction.

Recent studies have shown that age was a major determinant of SOS in both sexes. In females, SOS values had a much stronger correlation with age than male subjects [13]. Correspondingly, age and sex have a pronounced effect on the incidence rates of DRFs in the elderly population. Results of a large national registry of DRFs in adults showed that the vast majority of DRFs occurred in elderly women (≥ 50 years) [20]. Parallel to increasing age and decreasing estrogen, postmenopausal women experience loss and breakdown of bone mass [21]. The substantial increase in the number of fractures in postmenopausal women, and the ratio between women and men of 4:1, which could explain the lower bone strength of radius in women in our study [22]. Although it has been found in our research and similar studies that the SOS parameters of radius of men are more optimistic than women, it is worth noting that men over the age of 65 with DRFs are more likely to have post-fracture disability and fracture displacement and significantly associated with DRFs pattern complexity [23], indicating that there are also potential threats to the bone strength of the elderly male population [24]. Therefore, the assessment of future fracture risk among men with low radius SOS should not be neglected. Additionally, we found that the height of the low bone strength group significantly reduced compared with the normal group, which may be related to the damage of bone strength caused by osteoporosis [25, 26]. Height loss is a frequent manifestation of vertebral osteoporosis and is easy to measure in healthcare settings [27]. A significant association was observed between weight and bone strength which also corresponded to a previous study. It suggested that fat mass negatively correlated with BMD in young people [28]. Furthermore, integrating weight with age could modestly improve the prognosis of low bone strength in model 1 compared with the adjusted weight in model 2 because it is more sensitive and specific. Height and weight are also closely related to the range of BMI. A strong correlation between BMI and SOS parameters has been observed in diabetic patients [12]. Our findings are also indicated the correlation between BMI and SOS parameters; that is, higher BMI can predict lower bone strength of radius, an effect likely mediated by mechanical loading on bone [29]. In fact, fat mass and lean mass both cause mechanical loading on bone, and the relative effect of these two determinants of body composition on bone strength still remains controversial [29–31]. Logistic regression analysis showed that BMI was independent risk factors for DRFs [21], and a higher BMI increases the odds of a complex DRF [15]. However, others have shown that BMI does not affect the incidence of DRFs [32, 33], and other studies failed to detect the association between SOS or all three QUS measurements with BMI [34, 35]. In summary, we believe that changes in such indicators often cannot be understood separately, and the relation of SOS measures with BMI needs further investigation. Moreover, the nomograms as shown in this study are useful methods for communicating fractures risk to an individual patient, because they objectively incorporated many risk data of the individual patient.

The present findings should be interpreted within the context of some potential advantages and disadvantages. A major strength of the study is that the sample size was large, to allow for a reliable evaluation of relations between bone strength of radius and influencing factors. Moreover, this study includes both male and female populations across a broader age spectrum, which was quite rare in the same-topic study. In addition, SOS of radius provides relatively comprehensive bone strength information, while lower bone strength of radius is an independent risk factor for DRFs, which can provide a practical reference for clinical risk assessment of DRFs [6]. Nevertheless, our study has some limitations. First, SOS has some limitations due to the QUS device. For example, SOS results cannot be compared across devices, and the response to bone strength and ability to predict fractures of SOS is not as well studied as that of BMD. Additionally, this study was limited by its cross-sectional nature, with restriction of study cohort to only the Chinese population. However, the selection of grouping and modeling methods is fully based on the characteristics of the data, which provides a reference to other similar research. Although we used backward stepwise regression and LASSO regression to make models to compare which analysis method is better, there is only a slight difference between the two from the internal-verification effect of the model. Thus, external validation will be needed to clarify it further.

## Conclusions

In summary, our study demonstrates that the nomograms established in this study could effectively evaluate the risk of low distal bone strength in the Chinese population. Calibration plots, DCA, and ROC analysis verify the acceptable predictive value of the models. The nomograms may provide a referential basis for identifying the high-risk population groups of distal radius fracture.

## Data Availability

The datasets generated during and/or analyzed during the current study are available from the corresponding author on reasonable request.

## Abbreviations

SOS: Speed of sound
LBS: Low bone strength
DCA: Decision curve analysis
ROC: Receiver operating characteristics curve
BMD: Bone mineral density
QUS: Quantitative ultrasound
BMI: Body mass index
AUC: Area under the curve
CI: Confidence interval
NBS: Normal bone strength
DRFs: Distal radius fractures
